# Sodium-Glucose Cotransporter 2 Inhibitors for Patients With Prostate Cancer Undergoing Hormone Therapy

**DOI:** 10.1001/jamaoncol.2025.5869

**Published:** 2026-01-08

**Authors:** Ruofan Shi, Yongle Zhan, Ruochen Ma, Salida Ali, Chi Yao, Tsun Tsun Stacia Chun, Ada Tsui-Lin Ng, Matthew Kin-Liang Chiu, Bryan Cho Wing Li, Rong Na

**Affiliations:** 1 Division of Urology, Department of Surgery, School of Clinical Medicine, LKS Faculty of Medicine, The University of Hong Kong, Hong Kong, China; 2 Department of Urology, Ruijin Hospital, Shanghai Jiao Tong University School of Medicine, Shanghai, China; 3 Department of Family Medicine and Primary Care, LKS Faculty of Medicine, The University of Hong Kong, Hong Kong, China; 4 Division of Urology, Department of Surgery, Queen Mary Hospital, Hong Kong, China; 5 Department of Clinical Oncology, School of Clinical Medicine, LKS Faculty of Medicine, The University of Hong Kong, Hong Kong, China; 6 Centre of Cancer Medicine, School of Clinical Medicine, LKS Faculty of Medicine, The University of Hong Kong, Hong Kong, China

## Abstract

**Importance:**

While preliminary evidence suggests that sodium-glucose cotransporter 2 (SGLT2) inhibitors for diabetes may have antitumorigenic effects, their potential benefits in prostate cancer remain unexplored. Understanding their association with outcomes among patients undergoing hormone therapy could inform future adjunct treatment strategies.

**Objective:**

To evaluate whether the use of SGLT2 inhibitors is associated with clinical outcomes in patients with prostate cancer receiving hormone therapy.

**Design, Setting, and Participants:**

This population-based, sequential target trial emulation of monthly cohorts used territory-wide electronic health records (January 1, 1993, to April 30, 2025) from the Hong Kong Hospital Authority, covering a population of approximately 7.5 million. Adult men diagnosed with prostate cancer who initiated androgen deprivation therapy (ADT) were included. Follow-up extended through April 2025, and data were analyzed from June to October 2025.

**Exposures:**

Use of SGLT2 inhibitors (primarily dapagliflozin and empagliflozin) initiated during hormone therapy and maintained for at least 1 month. Comparator groups included nonusers of SGLT2 inhibitors.

**Main Outcomes and Measures:**

The primary outcome was time to ADT failure. Secondary outcomes include time to next-generation hormonal agent failure, disease-specific survival, and overall survival. Both intention-to-treat and per-protocol analyses were conducted using complementary log-log model regression to provide the hazard ratio (HR) estimate.

**Results:**

Among 14 223 eligible patients (median [IQR] age at enrollment, 74 [68-80] years) with a median follow-up of 66 months (95% CI, 65-67 months), intention-to-treat SGLT2 inhibitor use was associated with reduced risk of ADT failure (HR, 0.63; 95% CI, 0.41-0.95; *P* = .03) and next-generation hormonal agent failure (HR, 0.44; 95% CI, 0.20-0.97; *P* = .04). Sensitivity analyses confirmed robustness of these findings across different comparator subgroups. Metformin monotherapy was not associated with disease progression but was associated with improved overall survival (HR, 0.59; 95% CI, 0.42-0.83; *P* = .002). No statistically significant outcome differences were observed between dapagliflozin and empagliflozin.

**Conclusions and Relevance:**

In this cohort study with a target trial emulation design, SGLT2 inhibitor use was associated with delayed hormone therapy failure in patients with prostate cancer, suggesting a potential oncologic benefit beyond glucose lowering. These findings support the potential of SGLT2 inhibitors in treatment for prostate cancer.

## Key Points

**Question:**

Is use of antidiabetic agent sodium-glucose cotransporter 2 (SGLT2) inhibitors associated with clinical outcomes in patients with prostate cancer undergoing hormone therapy?

**Findings:**

In this cohort study with a target trial emulation design including 14 223 patients with prostate cancer, SGLT2 inhibitor use was associated with considerably lower risks of androgen deprivation therapy failure and lower risk of next-generation hormonal agent failure.

**Meaning:**

SGLT2 inhibitors may delay disease progression in patients with prostate cancer receiving hormone therapy, suggesting their potential role as adjuncts to hormonal therapy in the treatment of prostate cancer.

## Teaser

This cohort study evaluates whether the use of sodium-glucose cotransporter 2 (SGLT2) inhibitors is associated with clinical outcomes in patients with prostate cancer receiving hormone therapy.

## Introduction

Hormone therapy, including androgen deprivation therapy (ADT) and next-generation hormonal agents (NHAs), is a cornerstone that prolongs survival in advanced or recurrent prostate cancer by suppressing androgen-driven tumor growth.^1^ However, 45% to 75% of treated patients will develop drug resistance within 24 months.^2^ On the other hand, more than half of the patients receiving long-term ADT develop metabolic syndrome with symptoms like hyperglycemia and obesity,^3^ contributing to elevated risks of diabetes and cardiovascular disease. These phenomena underscore a need for therapeutic strategies that can address both oncologic control and metabolic health in patients with prostate cancer undergoing hormone therapy.

Sodium-glucose cotransporter 2 (SGLT2) inhibitors are antidiabetic medications originally developed to improve glycemic control by promoting urinary glucose excretion. Interestingly, emerging evidence suggests that SGLT2 inhibitors may also confer anticancer benefits beyond their metabolic effects.^4^ A recent study reported that initiating SGLT2 inhibitors in patients with prostate cancer was associated with prostate-specific antigen (PSA) responses, supporting further investigation of SGLT2 inhibitor use for prostate cancer.^5^ Another recent meta-analysis found that SGLT inhibitor use was associated with a lower overall risk of prostate cancer compared with dipeptidyl peptidase 4 inhibitors.^6^ An in vitro study also showed that SGLT2 blockade could inhibit prostate cancer cell proliferation, reduce invasion and metastasis, and enhance responses to radiotherapy.^7^ This evidence raises the possibility of repurposing SGLT2 inhibitors as adjunctive agents in prostate cancer management.

In the absence of randomized clinical trials (RCTs) evaluating this hypothesis, robust observational research is needed to evaluate the impact of SGLT2 inhibitors on prostate cancer outcomes. Target trial emulation (TTE) has emerged as a methodological framework to strengthen causal inference in observational studies, and dedicated reporting guidelines have further standardized its application. Recent studies have demonstrated its value in diverse clinical contexts, underscoring its potential to generate reliable evidence when RCTs are infeasible.^8,9^ In this study, we therefore applied a sequential TTE using electronic health records from the 7.5 million population in Hong Kong to investigate whether SGLT2 inhibitors may improve prognosis in patients with prostate cancer receiving hormone therapy, including ADT and NHAs. This framework mimics an RCT by explicitly aligning patient eligibility, treatment initiation, and follow-up timing in the observational dataset, thereby reducing biases such as immortal time and selection bias.^10^ Through this approach, we aimed to generate clinically relevant evidence on the potential effects of SGLT2 inhibitors in men undergoing hormone therapy for prostate cancer.

## Methods

### Study Design

This study used a sequential TTE design to evaluate the impact of SGLT2 inhibitors among patients with prostate cancer receiving hormone therapy. It was carried out on a monthly basis using electronic health records in the Clinical Data Analysis and Reporting System of the Hong Kong Hospital Authority from January 1, 1993, to April 30, 2025.

The study protocol was approved by the institutional review board of the University of Hong Kong/Hospital Authority Hong Kong West Cluster. Because all data involved in this study had been fully anonymized before extraction, patient informed consent was not required. This observational analysis was reported in accordance with the Strengthening the Reporting of Observational Studies in Epidemiology (STROBE) reporting guidelines^11^ and the recently published Transparent Reporting of Observational Studies Emulating a Target Trial (TARGET) guidelines^12^ to ensure transparent reporting.

### Study Population

Eligible patients were adult men diagnosed with prostate cancer (*International Classification of Diseases, Ninth Revision, Clinical Modification* code 185) who undertook ADT (eg, leuprorelin, goserelin, triptorelin, degarelix) with or without combined androgen blockade (CAB; eg, flutamide, bicalutamide). Exclusion criteria included (1) initiation of an NHA within 1 month after ADT/CAB initiation (ie, upfront NHA), (2) initiation of ADT/CAB within 3 months before or after radical prostatectomy (RP) with treatment duration less than 18 months, and (3) initiation of ADT/CAB within 3 months before or after radiotherapy with treatment duration of 6 months or less (ie, adjuvant ADT). We defined 3 subcohorts at baseline based on prescription records: (1) patients with prostate cancer who received SGLT2 inhibitors (ie, dapagliflozin, empagliflozin) at any time point during ADT, (2) patients with prostate cancer receiving glucose-lowering medications other than SGLT2 inhibitors (ie, alpha-glucosidase inhibitors, biguanides, dipeptidyl peptidase 4 inhibitors, glucagon-like peptide-1 receptor agonists, insulins, sulfonylureas, thiazolidinediones) at any time point during ADT, and (3) patients with prostate cancer receiving ADT with no diabetes medications.

### Intervention, Outcomes, and Follow-Up

The intervention of the current study was use of SGLT2 inhibitors during ADT. Patients were considered to have initiated the SGLT2 inhibitor intervention from the date of their initial prescription onward. Patients not receiving SGLT2 inhibitors (either those without diabetes or who received other diabetic medications except SGLT2 inhibitors) served as comparators. Continuous prescription episodes of SGLT2 inhibitors were merged into ongoing intervention intervals, allowing up to a 90-day grace period before considering the treatment interrupted.

The primary outcome of this study was time to ADT failure, which was defined as either biochemical progression (PSA rise ≥1.25 × the nadir and ≥2 ng/mL absolute increase [to convert to μg/L, multiply by 1], confirmed by 2 consecutive tests at least 2 weeks apart) or initiation of NHA (eg, abiraterone, enzalutamide, darolutamide, apalutamide) for disease progression. The secondary outcome was time to NHA failure, defined as the interval from the initiation of ADT to PSA progression with NHA initiation. The secondary outcomes also included disease-specific survival (DSS) and overall survival (OS).

Participants were followed up from the date of ADT initiation until the occurrence of an outcome event, death, the last visit to the hospital, or the administrative end date of the study (April 30, 2025), whichever came first. In Hong Kong, most patients with prostate cancer were followed up regularly at intervals of 3 to 6 months. In this study, all patients had recorded regular follow-up. During each follow-up, PSA levels were reevaluated, along with other clinical assessments when indicated. Patients who did not experience the outcome of interest were right censored at the time of their last follow-up.

### TTE

The specification and emulation of the target trials, following the TARGET statement,^12^ are described in eTable 1 in Supplement 1. This sequential TTE was implemented by initiating a new 1-month trial at the beginning of every calendar month (eFigure 1 in Supplement 1). At each monthly timeframe, eligibility criteria were reapplied and optimal pair-matched eligible individuals were enrolled into that month’s trial. Because the eligibility was reassessed monthly, the same person could contribute to several trials as long as they continued to meet the criteria, and robust variance estimators were applied to account for the resulting within-individual correlation.^13^ Those who initiated an SGLT2 inhibitor during the month of trial entry were assigned to the initiator group, while those who did not were classified as noninitiators. Two causal estimands were evaluated for every outcome: (1) the intention-to-treat (ITT) effect, where the participants remained in the group assigned at the trial baseline for the full duration of follow-up for that trial, and (2) the per-protocol (PP) effect, where follow-up was artificially censored at the first month in which observed treatment deviated from the group assigned at baseline.

### Statistical Analysis

Baseline clinical characteristics across subcohorts were compared using the Wilcoxon rank-sum test (2 groups) and Kruskal-Wallis rank-sum test (3 groups) for continuous variables, the Pearson χ^2^ test for categorical variables, and the log-rank test for survival outcomes. Median survival times were estimated using the Kaplan-Meier method.

For every monthly trial, propensity scores were estimated from baseline covariates (age, log-transformed PSA, and Charlson Comorbidity Index). Optimal pair matching was applied in a 1:4 ratio, using an exact match on calendar month of entry. When suitable matches were fewer than 4, the treated participant was retained with the available controls, so the final initiator to noninitiator ratio varied and may not be an integer.

At every month of follow-up, discrete-time hazard models with a complementary log-log link were fitted to the matched cohort, adjusting for age, PSA, Charlson Comorbidity Index, use of statins and nonstatin lipid-lowering drugs, use of cardiovascular medications (angiotensin-converting enzyme inhibitors, angiotensin II receptor blockers, angiotensin receptor-neprilysin inhibitors, antiplatelets, β-blockers, calcium channel blockers, and diuretics), metabolic and laboratory measures (glucose, hemoglobin A_1c_, high-density lipoprotein, low-density lipoprotein, total cholesterol, triglycerides, and kidney function), prior treatments (RP, radiotherapy, and chemotherapy), and socioeconomic indicators (occupational categories and residential district). All covariates were updated at each time point using a last observation carried forward approach. For variables with remaining missing values, multiple imputation by chained equations with predictive mean matching was applied. As the complementary log-log model is a discrete-time proportional hazards model, the exponentiated coefficients can be interpreted as hazard ratios (HRs),^14^ which we report in both ITT and PP analyses. In addition to HRs, we report absolute risks, absolute risk reductions, and numbers needed to treat at clinically relevant time points. Cumulative incidence curves were generated by predicting marginal cumulative risks under each treatment strategy. All modeling-related functions were from the R package TrialEmulation, version 0.0.4.2 (R Project for Statistical Computing).^13^

Prespecified analyses included comparisons across predefined subgroups within the comparator group, an evaluation of metformin monotherapy, a head-to-head comparison of dapagliflozin vs empagliflozin, and assessment of consistency between ITT and PP results. Additional sensitivity analyses were conducted by including the individuals receiving upfront NHA, varying the grace period for ADT and SGLT2 inhibitor prescriptions, and using conventional Cox regression models as a reference. All data assembly and analyses were conducted using R, version 4.4.2 (R Project for Statistical Computing).^15^ A -sided *P* < .05 was considered statistically significant. Data were analyzed from June to October 2025.

## Results

A total of 14 223 patients with prostate cancer (median [IQR] age at enrollment, 74 [68-80] years) who met the eligibility criteria were included in the analysis. The patient selection process is presented in eFigure 1 in Supplement 1, and patient baseline characteristics are summarized in eTable 2 in Supplement 1. The median follow-up was 66 months (95% CI, 65-67 months). A total of 6252 patients (44.0%) experienced ADT failure, with a median time to failure of 55 months (95% CI, 53-58 months). Among 3358 patients who received subsequent NHA, 1932 (57.5%) experienced treatment failure, with a median time to failure of 50 months (95% CI, 48-53 months). The prostate cancer–specific mortality was 16.8% (2384 deaths), and the overall mortality was 44.4% (6309 deaths). Before matching, certain imbalance of baseline characteristics across subgroups existed (eTable 2 in Supplement 1). After the propensity score matching, the baseline differences were attenuated (Table 1).

**Table 1.** Clinical Characteristics of the Study Population After Propensity Score Matching (PSM) by Treatment^a^

Characteristic	Median (IQR)		*P* value^b^	SMD	
	SGLT2 inhibitors (n = 159)	Others (n = 636)			
				Before PSM	After PSM
Age at enrollment, y	72 (66-76)	71 (67-76)	.79	−0.35	0.02
Serum total PSA before ADT, ng/mL	22.5 (9.5-92.2)	16.5 (6.1-59.7)	.02	−0.18	0.12
Baseline Charlson Comorbidity Index	3 (2-4)	3 (2-5)	.08	0.40	0.16

Abbreviations: ADT, androgen deprivation therapy; PSA, prostate-specific antigen; SGLT2, sodium-glucose cotransporter 2; SMD, standardized mean difference.

SI conversion factor: to convert PSA to μg/L, multiply by 1.

^a^ Matched population from the main analysis for the primary outcome.

^b^ A 2-sided *P* < .05 was considered statistically significant.

**Figure 1.**
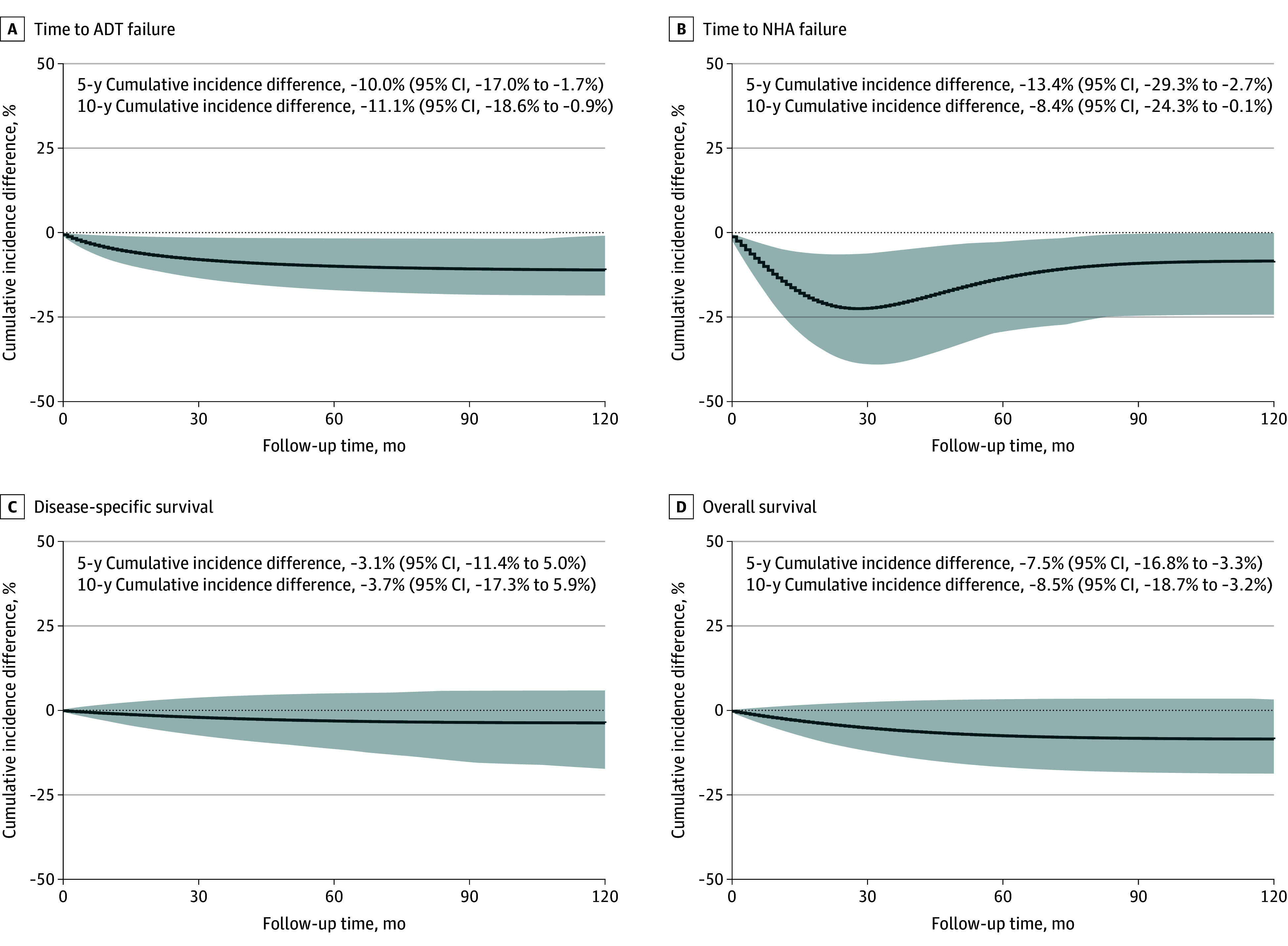
Cumulative Incidence Differences Between Users of Sodium-Glucose Cotransporter 2 Inhibitors and All Other Patients in the Intention-to-Treat Analysis Shaded areas represent 95% CIs. ADT indicates androgen deprivation therapy; NHA, next-generation hormonal agent.

In the ITT analysis (Table 2), receiving SGLT2 inhibitors was associated with statistically significant improved disease control while undergoing ADT compared to those who did not (time to ADT failure: HR, 0.63; 95% CI, 0.41-0.95; *P* = .03), resulting in a 11.1% decrease of the 10-year cumulative event rate (Figure 1A). Similarly, the use of SGLT2 inhibitors was associated with a statistically significant delayed time to failure of NHA (HR, 0.44; 95% CI, 0.20-0.97; *P* = .04), with an 8.4% decrease of the cumulative event rate at 10 years (Figure 1B). Although the associations with DSS and OS were not statistically significant, the point estimates suggested a favorable trend among those who used SGLT2 inhibitor (DSS: HR, 0.60; 95% CI, 0.20-1.85; *P* = .37; OS: HR, 0.70; 95% CI, 0.42-1.16; *P* = .17; Figure 1C and D). However, limited sample size of those taking NHAs (n = 215) and number of survival events (DSS, 41 events; OS, 123 events) resulted in low statistical power for detecting differences between groups (NHA analysis, 0.54; DSS analysis, 0.21; OS analysis, 0.31 [under the type I error of 0.05]). Similar findings were observed in the PP analysis (Table 2 and eFigure 2 in Supplement 1). The absolute risks, absolute risk reductions, and numbers needed to treat for ITT analyses are presented in eTable 3 in Supplement 1.

**Table 2.** Estimated Effects of Sodium-Glucose Cotransporter 2 (SGLT2) Inhibitors and Metformin on Treatment Failure and Survival Outcomes Among Patients With Prostate Cancer Undergoing Hormone Therapy

Intervention group	Control group	Causal estimand	Time to ADT failure			Time to NHA failure			Disease-specific survival			Overall survival		
			Intervention/ control, No.	HR (95% CI)	*P* value^a^	Intervention/ control, No.	HR (95% CI)	*P* value^a^	Intervention/ control, No.	HR (95% CI)	*P* value^a^	Intervention/ control, No.	HR (95% CI)	*P* value^a^
SGLT2 inhibitors	All others	ITT	159/636	0.63 (0.41-0.95)	.03	44/171	0.44 (0.20-0.97)	.04	160/640	0.60 (0.20-1.85)	.37	160/640	0.70 (0.42-1.16)	.17
		PP		0.60 (0.39-0.94)	.02		0.45 (0.18-1.12)	.08		0.68 (0.19-2.45)	.55		0.62 (0.34-1.13)	.12
	Nondiabetic	ITT	159/636	0.65 (0.43-1.00)	.05	44/157	0.39 (0.17-0.90)	.03	160/640	0.88 (0.39-2.00)	.76	160/640	0.95 (0.60-1.50)	.82
		PP		0.58 (0.36-0.94)	.03		0.40 (0.16-1.00)	.05		0.84 (0.29-2.42)	.74		0.89 (0.50-1.60)	.70
	Other diabetic drugs	ITT	159/592	0.62 (0.42-0.90)	.01	43/127	0.45 (0.20-0.99)	.04	160/601	0.54 (0.23-1.28)	.16	160/601	0.64 (0.41-0.99)	.04
		PP		0.58 (0.38-0.89)	.01		0.47 (0.19-1.15)	.10		0.46 (0.16-1.30)	.14		0.50 (0.29-0.85)	.01
Metformin monotherapy	Nondiabetic	ITT	293/1172	1.01 (0.76-1.33)	.96	45/180	1.37 (0.81-2.32)	.24	297/1185	0.79 (0.49-1.27)	.33	297/1185	0.59 (0.42-0.83)	.002
		PP		0.97 (0.71-1.33)	.87		0.85 (0.44-1.67)	.64		0.30 (0.14-0.64)	.002		0.35 (0.22-0.55)	<.001
Dapagliflozin	Empagliflozin	ITT	60/125	1.56 (0.81-3.00)	.19	NS	NS	NA	NS	NS	NA	60/125	0.33 (0.09-1.15)	.08
		PP		1.89 (0.90-3.97)	.09								0.23 (0.04-1.44)	.12

Abbreviations: ADT, androgen deprivation therapy; HR, hazard ratio; ITT, intention to treat; NHA, next-generation hormonal agent; NA, not applicable; NS, not stable; PP, per-protocol.

^a^ A 2-sided *P* < .05 was considered statistically significant.

**Figure 2.**
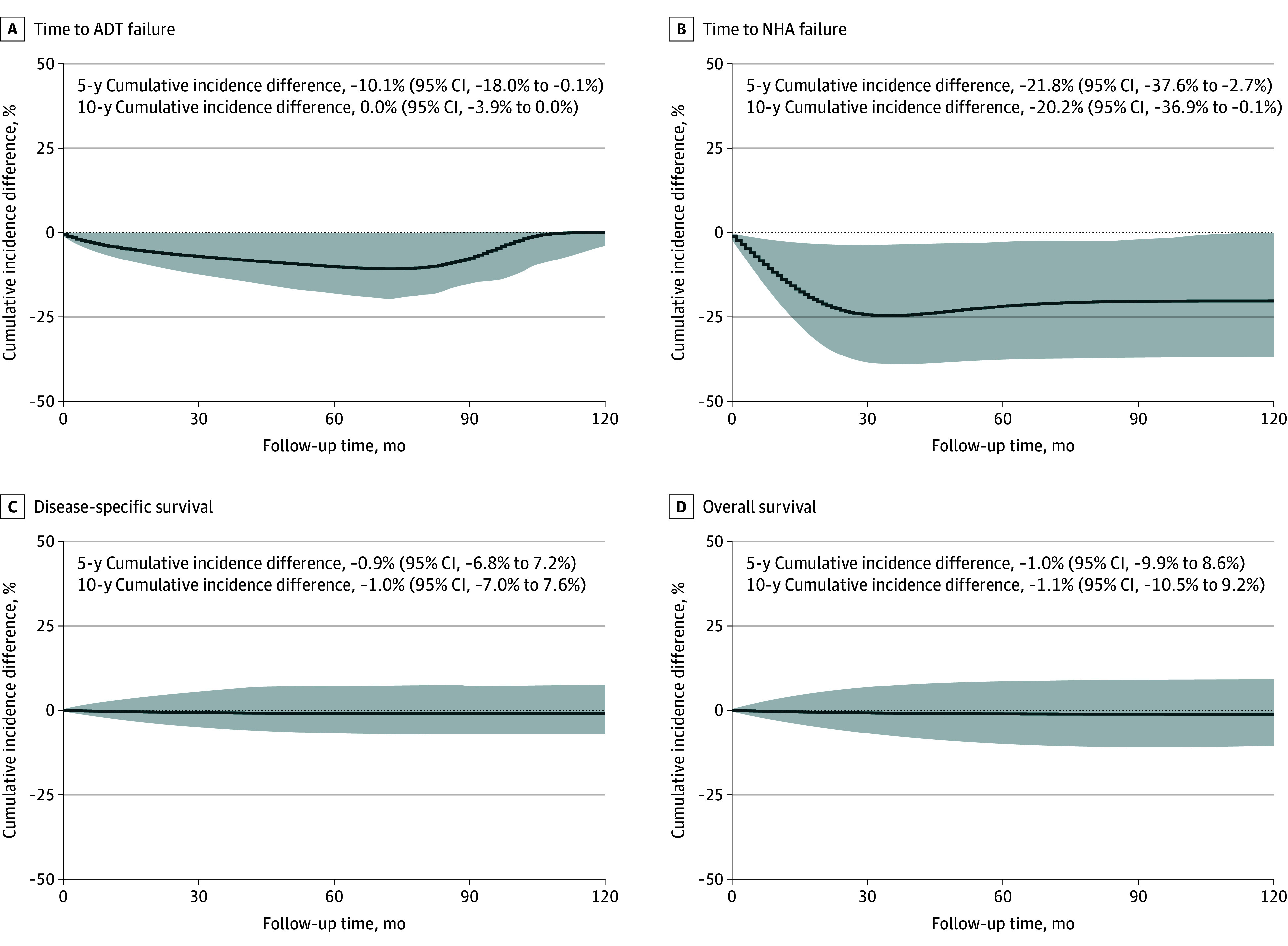
Cumulative Incidence Differences Between Users of Sodium-Glucose Cotransporter 2 Inhibitors and Patients Without Diabetes in the Intention-to-Treat Analysis Shaded areas represent 95% CIs. ADT indicates androgen deprivation therapy; NHA, next-generation hormonal agent.

**Figure 3.**
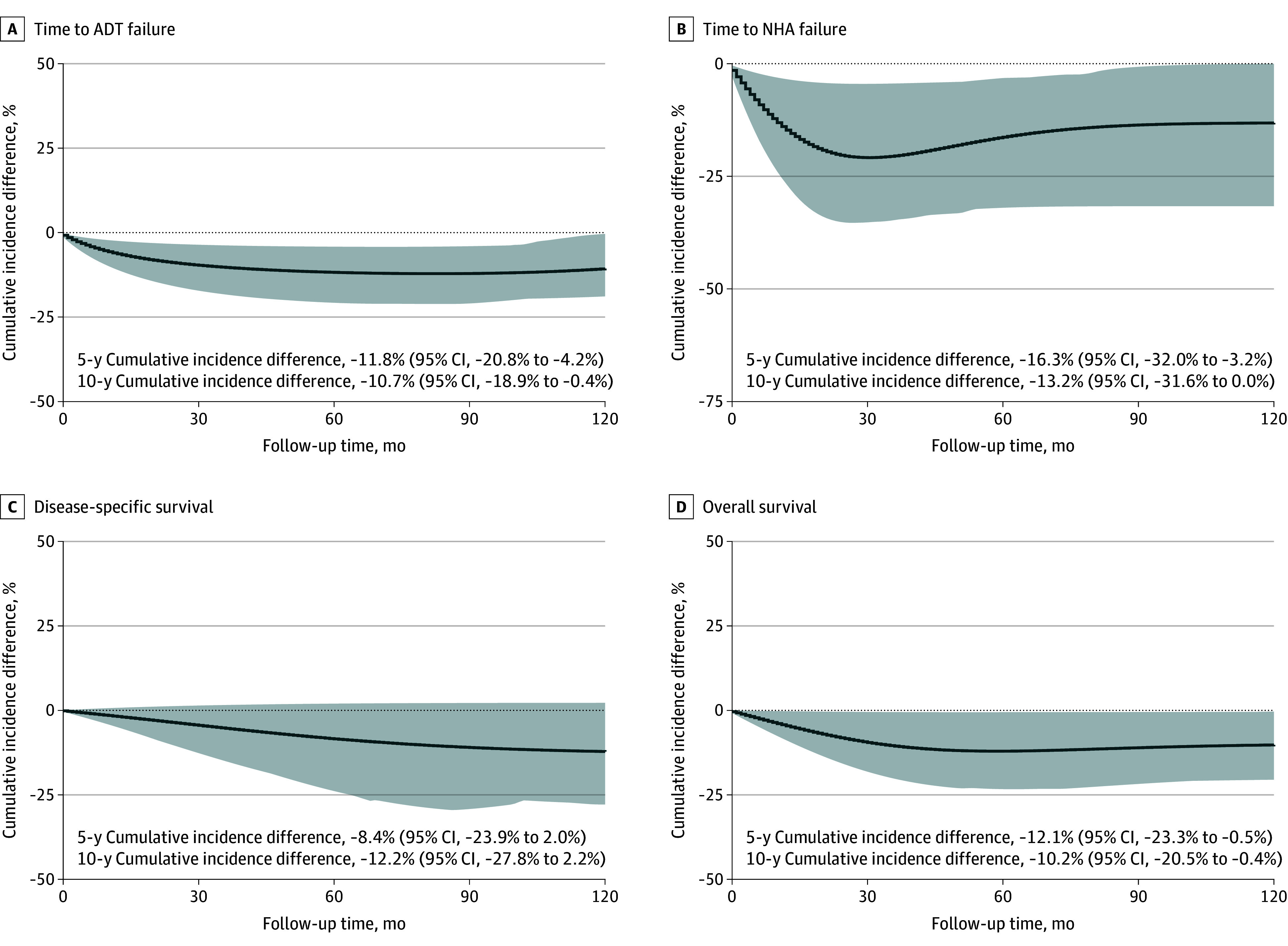
Cumulative Incidence Differences Between Users of Sodium-Glucose Cotransporter 2 Inhibitors and Patients Treated With Other Glucose-Lowering Drugs in the Intention-to-Treat Analysis Shaded areas represent 95% CIs. ADT indicates androgen deprivation therapy; NHA, next-generation hormonal agent.

Several sensitivity analyses were also conducted. Patients without diabetes and patients treated with other glucose-lowering drugs were further distinguished as comparator groups. Compared to patients without diabetes, those using SGLT2 inhibitors had a trend toward longer time to ADT failure (HR, 0.65; 95% CI, 0.43-1.00; *P* = .05; Table 2). A similar result was observed when compared with patients receiving other glucose-lowering agents (HR, 0.62; 95% CI, 0.42-0.90; *P* = .01). The associations with NHA failure and OS were also statistically significant in this analysis (NHA failure: HR, 0.45; 95% CI, 0.20-0.99; *P* = .04; OS: HR, 0.64; 95% CI, 0.41-0.99; *P* = .04; Table 2). The corresponding cumulative incidence differences are shown in Figures 2 and 3 (for ITT analysis) and eFigures 3 and 4 in Supplement 1 (for PP analysis).

Another subgroup analysis compared patients receiving metformin with patients without diabetes (Table 2 and eFigures 5 and 6 in Supplement 1). No statistically significant association was found between metformin use and ADT or NHA failure. However, improved OS was noted in this analysis (HR, 0.59; 95% CI, 0.42-0.83; *P* = .002). Considering that SGLT2 inhibitors are often prescribed together with metformin, this suggests that the SGLT2 inhibitors may have improved prostate cancer hormone therapy treatment outcomes via pathways other than glucose control, consistent with previous genetic findings.^16^ Subgroup analyses comparing dapagliflozin and empagliflozin did not reveal substantial differences in their associations with prostate cancer outcomes (Table 2 and eFigure 7 in Supplement 1).

Sensitivity analyses were generally consistent with the primary analyses (eTables 4-6 in Supplement 1). Supplementary Cox regression models confirmed robust associations across all outcomes (eTable 7 in Supplement 1).

## Discussion

Improving the outcomes of hormone therapy in patients with prostate cancer and reversing ADT/NHA resistance have been consistently a critical research topic with existing clinical gaps over the past decades. Some preliminary evidence based on in vitro experiments and genetic analysis suggested that SGLT2 inhibitors have a preventative effect on prostate cancer occurrences, providing rationale for this TTE.^16,17,18,19^ In this study, we found that patients with prostate cancer receiving SGLT2 inhibitors experienced statistically significant longer time to treatment failure of hormone therapy than those who did not use SGLT2 inhibitors. Furthermore, such potential treatment effect was not observed in patients taking other antidiabetic medications.

In subgroup analyses, the association between SGLT2 inhibition and delayed ADT failure held up across different patient strata. The beneficial trends were also observed when comparing patients receiving SGLT2 inhibitors to patients without diabetes or to patients taking other glucose-lowering drugs separately. This suggests that the possible effect may be linked to the SGLT2 inhibitors themselves rather than simply reflecting differences in underlying diabetes status. Meanwhile, we did not detect a substantial difference between dapagliflozin and empagliflozin in clinical outcomes, suggesting the consistent anti–prostate cancer effects within the class of SGLT2 inhibitors. As SGLT2 inhibitors are often used in combination with metformin, a sensitivity analysis of metformin monotherapy was conducted to further evaluate its effect. This analysis did not show any improvement in disease progression, which suggests that SGLT2 inhibitors may exert an independent protective effect during hormonal therapy for prostate cancer.

In vitro and preclinical studies have demonstrated that SGLT2 inhibitors can slow tumor growth in prostate cancer.^18^ For example, canagliflozin has been shown to suppress the proliferation of prostate cancer cells and to enhance their response to chemotherapy and radiotherapy, whereas dapagliflozin lacked such effects in the same models.^19^ Such anticancer activity provides a plausible mechanism for the delayed progression observed in patients treated with SGLT2 inhibitors in this study. Several biological mechanisms may underlie the apparent anticancer effects of SGLT2 inhibitors. For example, canagliflozin inhibits mitochondrial complex I, activates AMPK via increased adenosine monophosphate:adenosine triphosphate ratio, and subsequently suppresses mTOR-driven lipid synthesis and proliferation in cancer cells.^20^ Dapagliflozin can reduce oxidative stress via AMPK-SIRT1 signaling and diminish proinflammatory pathways.^21^ By lowering systemic inflammation and oxidative stress, SGLT2 inhibitors may further counteract the tumor-promoting microenvironment that can accompany diabetes and ADT. Although SGLT2 is expressed in various cancers, and blocking this transporter can directly reduce glucose uptake in tumor cells,^22^ a recent mendelian randomization analyses demonstrated a reduced prostate cancer risk with SGLT2 inhibitor exposure, while glycemic traits such as hemoglobin A_1c_ showed no meaningful association with prostate cancer incidence.^16^ Taken together with the present findings, these observations suggest that the glucose-centric hypothesis may fall short of explaining the antitumor effects of SGLT2 inhibition. Future biological experiments are needed to further evaluate this hypothesis and mechanism.

The interplay between diabetes and prostate cancer is complex and noteworthy. Meta-analyses show that men with long-standing diabetes have a modestly reduced risk of developing prostate cancer.^23^ However, men with diabetes who develop prostate cancer tend to have more aggressive disease. A study found that preexisting diabetes was linked to a 29% increase in prostate cancer–specific mortality and a 37% increase in overall mortality compared to those without diabetes.^24^ In other words, diabetes may shift the spectrum toward fewer but more lethal tumors. The present cohort likely reflects this interplay, which implies that an SGLT2 inhibitor–associated benefit on cancer progression was observed in patients with diabetes (with presumably higher risk for cancer). It remains possible that SGLT2 inhibitors could also impact tumor biology differentially in patients with vs without diabetes, which warrants further investigation in future studies.

Interestingly, metformin monotherapy did not confer benefit on ADT or NHA failure, but it was associated with improved OS in this study. This finding aligns with some of the prior observational studies and meta-analyses that reported survival advantages of metformin use among patients with prostate cancer.^25^ However, it contrasts with the recently published phase 3 STAMPEDE trial, which found no OS benefit when metformin was added to ADT among patients without diabetes.^26^ In the present study, the patients entered the target trial regardless of their diabetic status. Metformin is most commonly prescribed for type 2 diabetes. In the current analyses, the record of using metformin and diabetes diagnosis were concordant. In the STAMPEDE trial, one of the recruitment criteria was nondiabetic status. Whether patients with diabetes would benefit from the metabolic modulation from metformin seems debatable, and additional evidence is warranted.

### Limitations

The current study’s TTE design closely mimics an RCT and attenuates common biases of observational research. However, the number of patients who experienced NHA failure was relatively small after propensity score matching, limiting statistical power and yielding imprecise estimates of that end point. Similar to any nonrandomized study, residual confounding by unmatched factors cannot be excluded. In addition, the absence of Gleason score in the database precluded adjustment for tumor grade, an important prognostic determinant in prostate cancer. This limitation may have introduced residual heterogeneity in the cohort and highlights the need for validation in future studies with access to detailed pathological variables. Although we believe that treatment patterns (eg, RP, radiotherapy, chemotherapy) are highly relevant to disease stage, it cannot fully represent pathological staging. Nevertheless, this study suggests that SGLT2 inhibitors could plausibly serve as adjuncts to hormone therapy in patients with prostate cancer. These agents may help delay progression among those receiving hormone therapy while simultaneously improving metabolic health.

## Conclusions

This cohort study with a TTE design showed that the use of SGLT2 inhibitors among patients with prostate cancer was associated with delayed hormone therapy failure. However, cancer-specific and overall survival did not reach statistical significance. Prospective trials are warranted to validate these observations and assess their potential clinical applicability.

## Notes

Supplement 1. **eTable 1.** Specification and emulation of the target trial protocol **eFigure 1.** Participant selection flow diagram and schematic illustration of the sequential target trial emulation **eTable 2.** Clinical characteristics of the study population **eFigure 2.** Cumulative incidence difference between SGLT2 inhibitor users and all the others (PP) **eTable 3.** Absolute risks, absolute risk reduction, and numbers needed to treat of SGLT2 inhibitors and metformin vs comparators at different time points **eFigure 3.** Cumulative incidence difference between SGLT2 inhibitor users and non-diabetics (PP) **eFigure 4.** Cumulative incidence difference between SGLT2 inhibitor users and subjects treated with other glucose-lowering drugs (PP) **eFigure 5.** Cumulative incidence difference between metformin monotherapy and non-diabetics (ITT) **eFigure 6.** Cumulative incidence difference between metformin monotherapy and non-diabetics (PP) **eFigure 7.** Cumulative incidence difference between dapagliflozin and empagliflozin users **eTable 4.** Estimated effects of SGLT2 inhibitors and metformin on treatment failure and survival outcomes among prostate cancer patients undergoing hormone therapy (with upfront NHA included) **eTable 5. **Sensitivity analysis of SGLT2 inhibitors on treatment failure and survival outcomes among prostate cancer patients undergoing hormone therapy with an ADT grace period of 12 months **eTable 6.** Sensitivity analysis of SGLT2 inhibitors on treatment failure and survival outcomes among prostate cancer patients undergoing hormone therapy with varying SGLT2 inhibitor grace periods **eTable 7.** Sensitivity analyses of SGLT2 inhibitors on treatment failure and survival outcomes among prostate cancer patients undergoing hormone therapy using conventional Cox regression model

Supplement 2.
Data Sharing Statement
