# Supplementary material for: Sodium-Glucose Cotransporter 2 Inhibitors for Patients With Prostate Cancer Undergoing Hormone Therapy
Source: JAMA Oncol. 2026 Jan 8;12(3):257–65. doi: 10.1001/jamaoncol.2025.5869 (PMC12784257; doi:10.1001/jamaoncol.2025.5869)
Supplement: Supplement 1. — eTable 1. Specification and emulation of the target trial protocol eFigure 1. Participant selection flow diagram and schematic illustration of the sequential target trial emulation eTable 2. Clinical characteristics of the study population eFigure 2. Cumulative incidence difference between SGLT2 inhibitor users and all the others (PP) eTable 3. Absolute risks, absolute risk reduction, and numbers needed to treat of SGLT2 inhibitors and metformin vs comparators at different time points eFigure 3. Cumulative incidence difference between SGLT2 inhibitor users and non-diabetics (PP) eFigure 4. Cumulative incidence difference between SGLT2 inhibitor users and subjects treated with other glucose-lowering drugs (PP) eFigure 5. Cumulative incidence difference between metformin monotherapy and non-diabetics (ITT) eFigure 6. Cumulative incidence difference between metformin monotherapy and non-diabetics (PP) eFigure 7. Cumulative incidence difference between dapagliflozin and empagliflozin users eTable 4. Estimated effects of SGLT2 inhibitors and metformin on treatment failure and survival outcomes among prostate cancer patients undergoing hormone therapy (with upfront NHA included) eTable 5. Sensitivity analysis of SGLT2 inhibitors on treatment failure and survival outcomes among prostate cancer patients undergoing hormone therapy with an ADT grace period of 12 months eTable 6. Sensitivity analysis of SGLT2 inhibitors on treatment failure and survival outcomes among prostate cancer patients undergoing hormone therapy with varying SGLT2 inhibitor grace periods eTable 7. Sensitivity analyses of SGLT2 inhibitors on treatment failure and survival outcomes among prostate cancer patients undergoing hormone therapy using conventional Cox regression model [file jamaoncol-e255869-s001.pdf]

Supplemental Online Content

Shi R, Zhan Y, Ma R, et al. Sodium-glucose cotransporter 2 inhibitors for patients with prostate cancer undergoing hormone therapy. *JAMA Oncol*. Published online January 8, 2026. doi:10.1001/jamaoncol.2025.5869

eTable 1. Specification and emulation of the target trial protocol
eFigure 1. Participant selection flow diagram and schematic illustration of the sequential target trial emulation
eTable 2. Clinical characteristics of the study population
eFigure 2. Cumulative incidence difference between SGLT2 inhibitor users and all the others (PP)
eTable 3. Absolute risks, absolute risk reduction, and numbers needed to treat of SGLT2 inhibitors and metformin vs comparators at different time points
eFigure 3. Cumulative incidence difference between SGLT2 inhibitor users and non-diabetics (PP)
eFigure 4. Cumulative incidence difference between SGLT2 inhibitor users and subjects treated with other glucose-lowering drugs (PP)
eFigure 5. Cumulative incidence difference between metformin monotherapy and non-diabetics (ITT)
eFigure 6. Cumulative incidence difference between metformin monotherapy and non-diabetics (PP)
eFigure 7. Cumulative incidence difference between dapagliflozin and empagliflozin users
eTable 4. Estimated effects of SGLT2 inhibitors and metformin on treatment failure and survival outcomes among prostate cancer patients undergoing hormone therapy (with upfront NHA included)
eTable 5. Sensitivity analysis of SGLT2 inhibitors on treatment failure and survival outcomes among prostate cancer patients undergoing hormone therapy with an ADT grace period of 12 months
eTable 6. Sensitivity analysis of SGLT2 inhibitors on treatment failure and survival outcomes among prostate cancer patients undergoing hormone therapy with varying SGLT2 inhibitor grace periods
eTable 7. Sensitivity analyses of SGLT2 inhibitors on treatment failure and survival outcomes among prostate cancer patients undergoing hormone therapy using conventional Cox regression model

This supplemental material has been provided by the authors to give readers additional information about their work.

© 2026 Shi R et al. *JAMA Oncol.*

eTable 1. Specification and emulation of the target trial protocol

Protocol Component	Target Trial	Observational Emulation
**Eligibility Criteria**	Men (over 18 years) with confirmed PCa, initiating ADT; no prior SGLT2 inhibitors use before trial enrollment.	Same as the target trial.<br>The eligibility is assessed at monthly intervals during the study period. Each patient can contribute to multiple sequential trials.
**Treatment Strategies**	Intervention: Initiate an SGLT2 inhibitor in addition to ongoing ADT.<br>Comparator: Do not initiate any SGLT2 inhibitors (ADT alone). All other aspects of management are as per standard care in both arms.	Intervention: Same as the target trial.<br>Comparator: Same as the target trial.<br>Standard care is matched to be similar between groups aside from SGLT2 inhibitors use.
**Assignment Procedures**	Eligible participants are randomized to either the intervention or comparator group at the time of ADT initiation. Randomization is open-label, but allocation is by chance.	Group assignment is determined by the observed treatment in the prescription records at the baseline of each emulated trial. To emulate randomization, each monthly trial's new entrants are similarly assigned the analysis adjusts for baseline covariates using optimal pair matching.
**Follow-up**	Follow-up begins at the point of randomization, which coincides with SGLT2 inhibitor initiation for the intervention group (or the equivalent time for the control group).<br>Follow-up ends at the earliest of: PCa progression (outcomes occurrence), death, loss to follow-up, or a fixed end of trial. All participants are followed for the occurrence of outcome or censoring; outcome ascertainment is blinded to assignment in the target trial.	Follow-up starts at the time of treatment decision in each emulated trial, which corresponds to time zero for each monthly cohort.<br>Same as the target trial.<br>In practice, progression and survival status are tracked via EHR. Importantly, each monthly-start cohort has its own follow-up clock, and all are pooled in the final analysis.
**Causal Contrasts**	Compare the effect of sustained use of SGLT2 inhibitors versus no sustained use among PCa patients initiating ADT. The primary effect measure was the HR for time to ADT failure; AR and ARR at prespecified follow-up times were also considered.	Same as the target trial.<br>In practice, sustained SGLT2 inhibitor use is defined with a 90-day grace period (with sensitivity analyses using alternative grace periods). Outcomes are estimated using complementary log-log (cloglog) regression to yield HRs, with Cox regression performed as a sensitivity analysis. AR, ARR, and NNT are also reported.

© 2026 Shi R et al. *JAMA Oncol.*

		Same as the target trial.
**Identifying Assumptions**	Exchangeability is guaranteed by randomization.<br>Consistency holds under well-defined treatment strategies.<br>Positivity is ensured because each eligible patient has a non-zero probability of assignment to either strategy.<br>Non-informative censoring is assumed.	Because treatment is not randomized, conditional exchangeability is assumed after adjusting for measured baseline confounders (age, PSA, comorbidity, etc.) through optimal pair matching and adjustment in the outcome model. Positivity is assumed for all covariate strata. Consistency is assumed under the definition of sustained SGLT2i use with a 90-day grace period. Non-informative censoring is assumed. Sensitivity analyses with alternative grace periods and model specifications (Cox) are used to assess robustness.
**Outcomes**	Primary outcome: time to ADT failure.<br>Secondary outcomes: time to NHA failure, PCa-specific survival and overall survival.	Same as the target trial.
**Data Analysis**	Use survival analysis (Kaplan-Meier curves and log-rank test) to compare the ITT and PP effect between arms. Use Cox proportional hazards models to estimate the HR for the outcomes, with the corresponding 95% CI. Predefined subgroup analyses would be performed to explore effect modification.	A complementary log-log regression is used as equivalent to estimate the HRs under both ITT and PP analysis. This model is weighted for time-varying covariates. Robust standard errors account for patients who appear in multiple sequential trials, ensuring correct variance estimation. Sensitivity analyses were also performed to test the robustness of findings.

Abbreviations: PCa: prostate cancer; ADT: androgen deprivation therapy; SGLT2: sodium-glucose cotransporter 2; NHA: next-generation hormonal agent; EHR: electronic health record; HR: hazard ratio; AR: absolute risk; ARR: absolute risk reduction; NTT: numbers needed to treat; PSA: prostate-specific antigen; ITT: intention-to-treat; PP: per-protocol; 95% CI: 95% confidence interval.

© 2026 Shi R et al. *JAMA Oncol.*

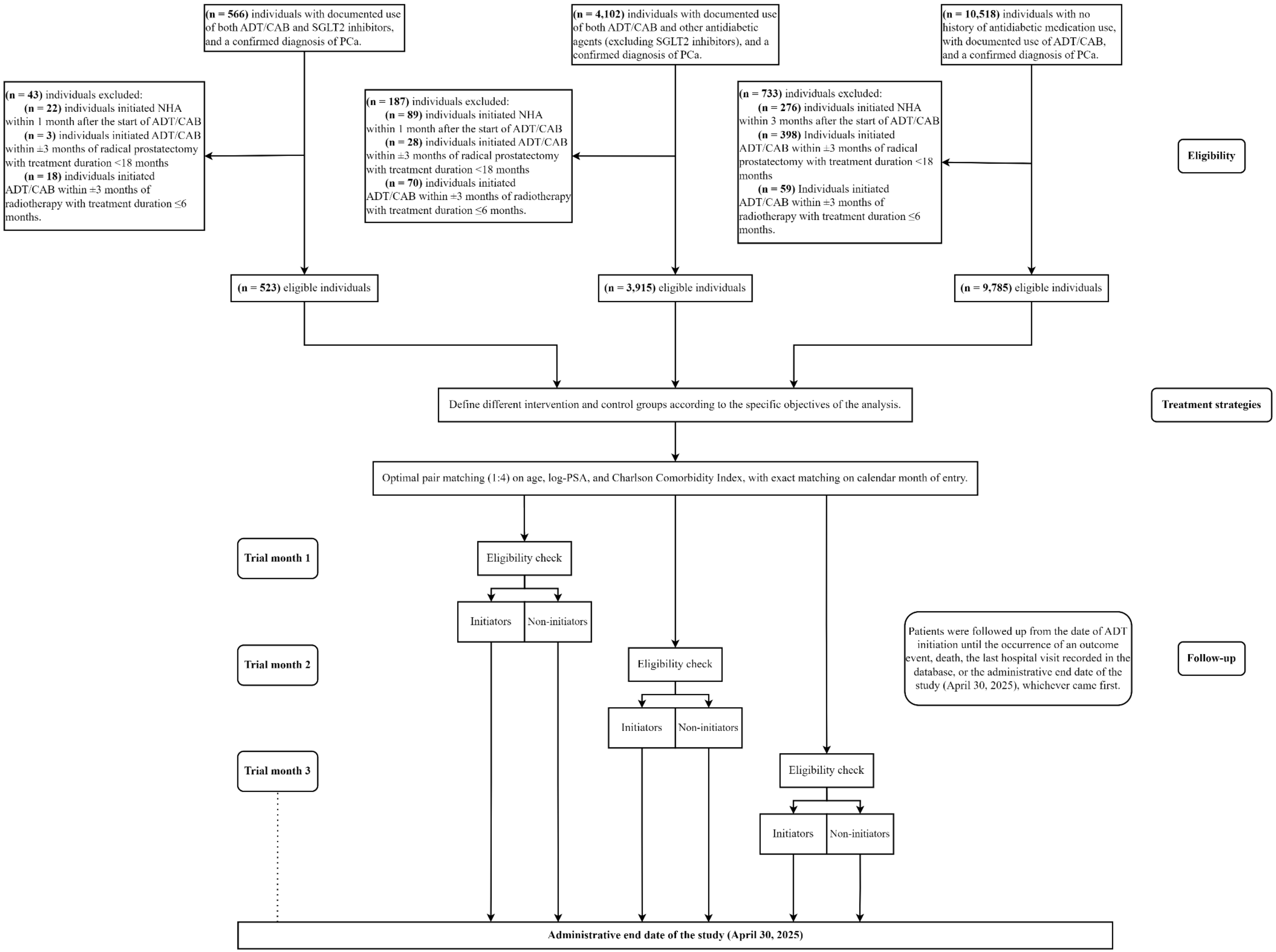

© 2026 Shi R et al. *JAMA Oncol.*

eFigure 1. Participant selection flow diagram and schematic illustration of the sequential target trial emulation

Patients with PCa receiving ADT/CAB were included, while those with early NHT, peri-RP or peri-RT ADT/CAB of short duration were excluded. Sequential monthly trials were emulated, with optimal pair matching (1:4) on age, log-PSA, and CCI, and exact matching on calendar month. Follow-up continued until outcome, death, last hospital visit, or April 30, 2025.

Abbreviations: ADT: androgen deprivation therapy; CAB: combined androgen blockade; SGLT2: sodium-glucose cotransporter 2; PCa: prostate cancer; NHA: next-generation hormonal agent; RP: radical prostatectomy; RT: radiotherapy; PSA: prostate-specific antigen; CCI: Charlson Comorbidity Index.

© 2026 Shi R et al. *JAMA Oncol.*

eTable 2. Clinical characteristics of the study population

Characteristics	Overall population (*N*=14,223)	SGLT2 inhibitors (*n*=523)	Non-diabetic (*n*=9,785)	Other diabetic drugs (*n*=3,915)	*P*
Age at enrolment (years), median (IQR)	74 (68-80)	72 (67-78)	74 (68-81)	75 (69-80)	**<.001**
Serum total PSA before ADT (ng/mL), median (IQR)	37.7 (11.1-182.9)	30.2 (10.6-121.2)	34.0 (10.6-173.0)	49.9 (12.7-210.8)	**<.001**
Baseline Charlson Comorbidity Index, median (IQR)	2 (2-4)	3 (1-4)	2 (2-4)	3 (2-5)	**<.001**
Mediam follow-up time (months), median (95% CI)	66 (65-67)	42 (39-48)	63 (61-65)	82 (79-85)	**<.001**
No. of ADT failure, *n* (%)	6,252 (44.0)	203 (38.8)	4,053 (41.4)	1,996 (51.0)	**<.001**
Median time to ADT failure (months), median (95% CI)	55 (53-58)	66 (55-83)	63 (59-66)	41 (38-45)	**<.001**
No. of NHA failure, *n*/*N* (%)	1,932/3,358 (57.5)	62/132 (47.0)	1,184/2,135 (55.5)	686/1,091 (62.9)	**<.001**
Median time to NHA failure (months), median (95% CI)	50 (48-53)	54 (46-79)	51 (48-54)	49 (45-53)	.10
No. of PCa-specific death, *n* (%)	2,384 (16.8)	42 (8.0)	1,510 (15.4)	823 (21.3)	**<.001**
No. of all-cause death, *n* (%)	6,309 (44.4)	114 (21.8)	4,063 (41.5)	2,132 (54.5)	**<.001**
Median survival time (months), median (95% CI)	80 (78-83)	134 (111-N/A)	85 (83-88)	67 (64-71)	**<.001**

Abbreviations: SGLT2: sodium-glucose cotransporter 2; IQR: interquartile range; PSA: prostate-specific antigen; ADT: androgen deprivation therapy; 95% CI: 95% confidence interval; NHA: next-generation hormonal agent; PCa: prostate cancer; N/A: not applicable. A two-sided *P*-value <0.05 was considered statistically significant.

© 2026 Shi R et al. *JAMA Oncol.*

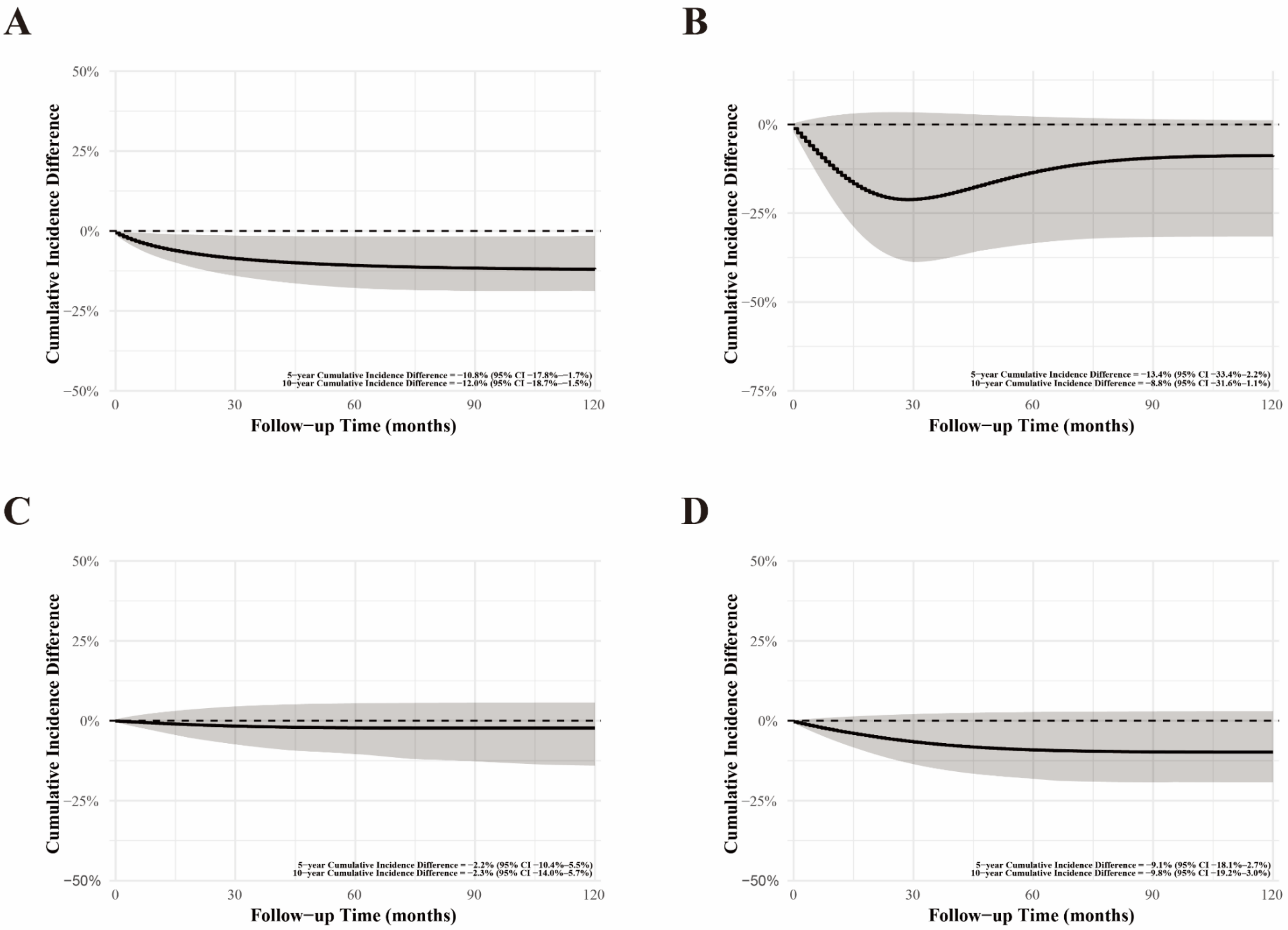

© 2026 Shi R et al. *JAMA Oncol.*

eFigure 2. Cumulative incidence difference between SGLT2 inhibitor users and all the others (PP)

The difference in cumulative incidence for SGLT2 inhibitor users relative to all the other subjects in the PP analysis. (**A**) time to ADT failure; (**B**) time to NHA failure; (**C**) disease-specific survival; (**D**) overall survival.

Abbreviations: SGLT2: sodium-glucose cotransporter 2; PP: per-protocol; 95% CI: 95% confidence interval.

© 2026 Shi R et al. *JAMA Oncol.*

eTable 3. Absolute risks, absolute risk reduction, and numbers needed to treat of SGLT2 inhibitors and metformin vs comparators at different time points†

Intervention Group (I)	Control Group (C)	Time to ADT Failure						Time to NHA Failure					
		Risk Set (I/C)	Time point (months)	AR - Intervention (%)	AR - Control (%)	ARR (%, 95% CI)	NNT (95% CI)	Risk Set (I/C)	Time point (months)	AR - Intervention (%)	AR - Control (%)	ARR (%, 95% CI)	NNT (95% CI)
SGLT2 inhibitors	All the others	97/352	12	11.2 (9.4-16.8)	16.3 (15.0-18.9)	5.1 (-0.6-7.9)	20 (-Inf-Inf)	34/127	12	16.8 (11.0-29.0)	32.2 (28.0-38.1)	15.4 (3.9-25.0)	7 (4-26)
		52/190	24	18.2 (15.1-26.1)	25.5 (22.7-29.4)	7.3 (-0.8-11.2)	14 (-Inf-Inf)	15/50	24	36.8 (25.1-53.2)	59.0 (52.6-65.9)	22.2 (5.0-36.4)	5 (3-21)
		29/100	36	23.5 (19.4-33.1)	32.1 (28.2-38.3)	8.6 (-0.9-13.5)	12 (-Inf-Inf)	6/22	36	55.0 (37.9-70.1)	76.2 (68.2-82.7)	21.2 (4.5-37.5)	5 (3-23)
		6/17	60	31.6 (26.0-43.4)	41.5 (34.3-52.6)	10.0 (-1.0-16.0)	11 (-Inf-Inf)	1/2	60	75.8 (53.7-89.3)	89.2 (79.3-93.7)	13.4 (2.3-29.7)	8 (4-44)
	Non-diabetic	97/373	12	10.5 (8.8-14.8)	14.9 (13.8-17.2)	4.4 (0.3-6.7)	23 (16-317)	34/121	12	13.3 (9.0-24.5)	28.1 (24.4-33.5)	14.8 (2.3-22.1)	7 (5-45)

© 2026 Shi R et al. *JAMA Oncol.*

		52/194	24	16.9 (14.6-22.7)	23.3 (20.9-27.9)	6.4 (0.4-9.6)	16 (11-227)	15/50	24	29.1 (19.2-46.1)	52.1 (44.3-59.5)	23.0 (3.1-34.1)	5 (3-32)
		29/100	36	22.7 (19.5-30.2)	30.4 (26.5-37.2)	7.8 (0.5-11.8)	13 (9-190)	6/21	36	43.5 (28.8-60.8)	68.1 (57.9-75.1)	24.6 (3.1-37.4)	5 (3-33)
		6/20	60	38.2 (31.2-54.0)	48.3 (38.9-62.9)	10.1 (0.6-15.7)	10 (7-159)	1/3	60	59.6 (39.6-79.9)	81.4 (68.0-89.6)	21.8 (2.7-35.9)	5 (3-38)
		97/348	12	13.1 (9.7-17.4)	19.4 (17.6-22.2)	6.3 (2.0-11.3)	16 (9-51)	33/88	12	18.2 10.6-32.0)	33.0 (29.8-39.7)	14.8 (0.3-26.3)	7 (4-302)
	Other diabetic drugs	52/196	24	21.3 (16.1-27.3)	30.1 (26.1-34.9)	8.9 (2.7-16.2)	12 (7-37)	15/34	24	36.0 (21.4-54.1)	56.3 (48.6-64.0)	20.3 (0.4-36.9)	5 (3-263)
		29/93	36	27.7 (20.7-35.9)	38.0 (31.8-44.5)	10.3 (3.1-19.3)	10 (6-33)	6/19	36	51.0 (31.4-69.3)	71.4 (61.4-77.5)	20.5 (0.4-38.5)	5 (3-277)
		6/14	60	38.4 (29.4-53.2)	50.2 (39.6-61.4)	11.8 (3.4-22.8)	9 (5-30)	1/2	60	68.5 (43.3-86.0)	84.8 (72.4-93.4)	16.3 (0.3-34.8)	7 (3-381)
Metformin monotherapy	Non-diabetic	212/768	12	13.1 (11.3-15.7)	13.0 (12.0-14.3)	-0.1 (-	-1,439 (-	33/117	12	22.9 (17.8-31.1)	17.9 (15.6-23.0)	-5.1 (-	-19 (-Inf-Inf)

© 2026 Shi R et al. *JAMA Oncol.*

						2.9-2.0)	Inf-Inf)					13.9 -4.7)	
		146/485	24	21.2 (18.5-25.3)	21.1 (19.3-23.0)	-0.1 (-4.2-3.0)	-977 (-Inf-Inf)	15/72	24	37.0 (29.2-46.8)	30.0 (25.7-36.8)	-7.1 (-18.0 -6.4)	-14 (-Inf-Inf)
		92/346	36	27.4 (23.8-32.6)	27.3 (24.9-30.0)	-0.1 (-5.0-3.6)	-814 (-Inf-Inf)	11/42	36	47.5 (38.1-57.7)	39.6 (33.1-47.9)	-8.0 (-19.6 -7.1)	-12 (-Inf-Inf)
		48/168	60	36.9 (31.6-43.5)	36.8 (33.3-40.8)	-0.1 (-6.0-4.3)	-681 (-Inf-Inf)	4/20	60	62.7 (50.5-73.5)	54.4 (43.4-64.5)	-8.3 (-19.1 -7.1)	-12 (-Inf-Inf)
Dapagliflozin	Empagliflozin	36/90	12	15.1 (12.3-22.2)	10.8 (9.0-16.2)	-4.3 (-12.9 -1.6)	-23 (-Inf-Inf)	Results not stable.					
		19/58	24	22.5 (17.2-31.1)	16.7 (13.1-24.5)	-5.9 (-16.6 -2.0	-17 (-Inf-Inf)						
		9/20	36	27.8 (20.6-38.8)	21.1 (15.3-32.3)	-6.7 (-18.2 -2.2)	-14 (-Inf-Inf)						
		3/3	60	35.9 (23.5-60.9)	28.2 (16.7-55.3)	-7.7 (-19.4 -2.5)	-13 (-Inf-Inf)						

© 2026 Shi R et al. *JAMA Oncol*.

Intervention Group (I)	Control Group (C)	Disease-specific Survival						Overall Survival					
		Risk Set (I/C)	Time point (months)	AR - Intervention (%)	AR - Control (%)	ARR (%, 95% CI)	NNT (95% CI)	Risk Set (I/C)	Time point (months)	AR - Intervention (%)	AR - Control (%)	ARR (%, 95% CI)	NNT (95% CI)
SGLT2 inhibitors	All the others	129/437	12	1.9 (1.1-4.3)	3.0 (2.8-5.4)	1.1 (-0.6-3.6)	94 (-Inf-Inf)	129/437	12	7.6 (5.4-11.9)	10.3 (8.9-12.9)	2.7 (-1.2-6.0)	38 (-Inf-Inf)
		78/252	24	3.5 (2.1-7.4)	5.3 (4.4-9.0)	1.8 (-0.9-6.0)	56 (-Inf-Inf)	78/252	24	14.3 (10.3-21.3)	18.8 (15.4-24.0)	4.5 (-2.0-10.1)	23 (-Inf-Inf)
		41/128	36	4.8 (3.3-10.0)	7.2 (5.8-12.8)	2.4 (-1.1-8.1)	43 (-Inf-Inf)	41/128	36	20.5 (15.4-30.1)	26.4 (21.4-33.7)	5.9 (-2.6-13.4)	18 (-Inf-Inf)
		8/25	60	6.8 (4.7-15.6)	10.0 (7.2-20.4)	3.1 (-1.3-11.5)	32 (-Inf-Inf)	8/25	60	30.4 (22.6-43.9)	37.9 (28.5-51.5)	7.5 (-3.2-17.7)	14 (-Inf-Inf)
	Non-diabetic	129/457	12	2.9 (1.8-6.6)	3.2 (3.1-5.2)	0.3 (-2.6-2.8)	299 (-Inf-Inf)	129/457	12	8.4 (6.4-12.9)	8.8 (8.0-10.9)	0.4 (-4.1-3.2)	260 (-Inf-Inf)

© 2026 Shi R et al. *JAMA Oncol*.

	78/257	24	5.3 (3.3-10.8)	5.8 (5.4-9.3)	0.6 (-4.2-4.8)	177 (-Inf-Inf)	78/257	24	15.7 (12.3-23.6)	16.3 (14.2-20.5)	0.6 (-6.4-5.5)	155 (-Inf-Inf)
	41/130	36	7.2 (4.8-14.8)	7.9 (7.1-12.9)	0.7 (-5.3-6.1)	137 (-Inf-Inf)	41/130	36	22.1 (17.5-33.8)	22.9 (19.4-29.5)	0.8 (-7.8-7.2)	121 (-Inf-Inf)
	8/28	60	9.4 (6.0-24.6)	10.3 (8.8-23.1)	0.9 (-6.6-7.8)	110 (-Inf-Inf)	8/28	60	31.1 (23.5-51.5)	32.2 (26.8-47.0)	1.0 (-9.8-9.2)	98 (-Inf-Inf)
Other diabetic drugs	129/429	12	2.4 (1.4-5.8)	4.2 (4.1-6.8)	1.8 (-1.1-4.8)	56 (-Inf-Inf)	129/429	12	10.0 (8.2-15.5)	14.5 (13.5-17.9)	4.6 (0.1-8.7)	22 (12-804)
	78/264	24	5.2 (3.3-10.3)	8.7 (8.0-14.4)	3.6 (-1.8-10.0)	29 (-Inf-Inf)	78/264	24	20.9 (17.3-30.2)	29.0 (25.8-35.7)	8.1 (0.2-15.0)	13 (7-459)
	41/135	36	8.4 (5.5-16.0)	13.7 (11.5-23.8)	5.3 (-2.4-15.5)	19 (-Inf-Inf)	41/135	36	32.3 (26.2-45.3)	42.9 (36.9-52.7)	10.6 (0.3-19.1)	10 (6-348)
	8/26	60	15.6 (9.2-30.1)	24.0 (15.4-44.1)	8.4 (-3.0-25.7)	12 (-Inf-Inf)	8/26	60	52.0 (40.3-71.7)	64.0 (52.5-78.0)	12.1 (0.5-23.3)	9 (5-298)

© 2026 Shi R et al. *JAMA Oncol*.

Metformin monotherapy	Non-diabetic	244/896	12	3.0 (2.2-4.4)	3.6 (3.5-4.6)	0.7 (-0.6-1.7)	148 (-Inf-Inf)	244/896	12	6.5 (5.4-8.3)	10.2 (9.3-11.6)	3.7 (1.5-5.6)	28 (18-67)
		178/640	24	5.3 (4.1-7.8)	6.4 (6.0-8.1)	1.1 (-1.0-3.0)	89 (-Inf-Inf)	178/640	24	12.1 (10.2-15.0)	18.2 (16.8-20.4)	6.1 (2.5-9.2)	17 (11-41)
		120/465	36	7.4 (5.8-10.8)	8.9 (8.2-11.3)	1.5 (-1.3-4.1)	68 (-Inf-Inf)	120/465	36	17.6 (14.9-21.5)	25.6 (23.6-28.8)	8.0 (3.2-11.9)	13 (9-32)
		67/252	60	11.0 (8.7-15.8)	13.1 (11.5-16.8)	2.0 (-1.8-5.8)	49 (-Inf-Inf)	67/252	60	28.0 (23.8-34.1)	38.7 (35.0-44.7)	10.7 (4.2-15.6)	10 (7-24)
Dapagliflozin	Empagliflozin	Results not stable.						49/106	12	5.2 (2.6-14.0)	11.3 (9.3-16.9)	6.2 (-1.1-10.8)	17 (-Inf-Inf)
								29/73	24	13.4 (7.6-29.0)	25.3 (19.9-34.0)	11.9 (-1.8-20.1)	9 (-Inf-Inf)
								13/30	36	24.7 (16.1-43.9)	39.6 (30.6-48.3)	15.0 (-1.9-25.1)	7 (-Inf-Inf)

© 2026 Shi R et al. *JAMA Oncol*.

	4/5	60	45.1 (30.6-59.4)	57.3 (42.0-65.4)	12.2 (-1.8-21.6)	9 (-Inf-Inf)

† From intention-to-treat analysis only.

Abbreviations: SGLT2: sodium-glucose cotransporter 2; ADT: androgen deprivation therapy; NHA: next-generation hormonal agent; AR: absolute risk; ARR: absolute risk reduction; 95% CI: 95% confidence interval; NTT: numbers needed to treat; Inf: infinity.

© 2026 Shi R et al. *JAMA Oncol.*

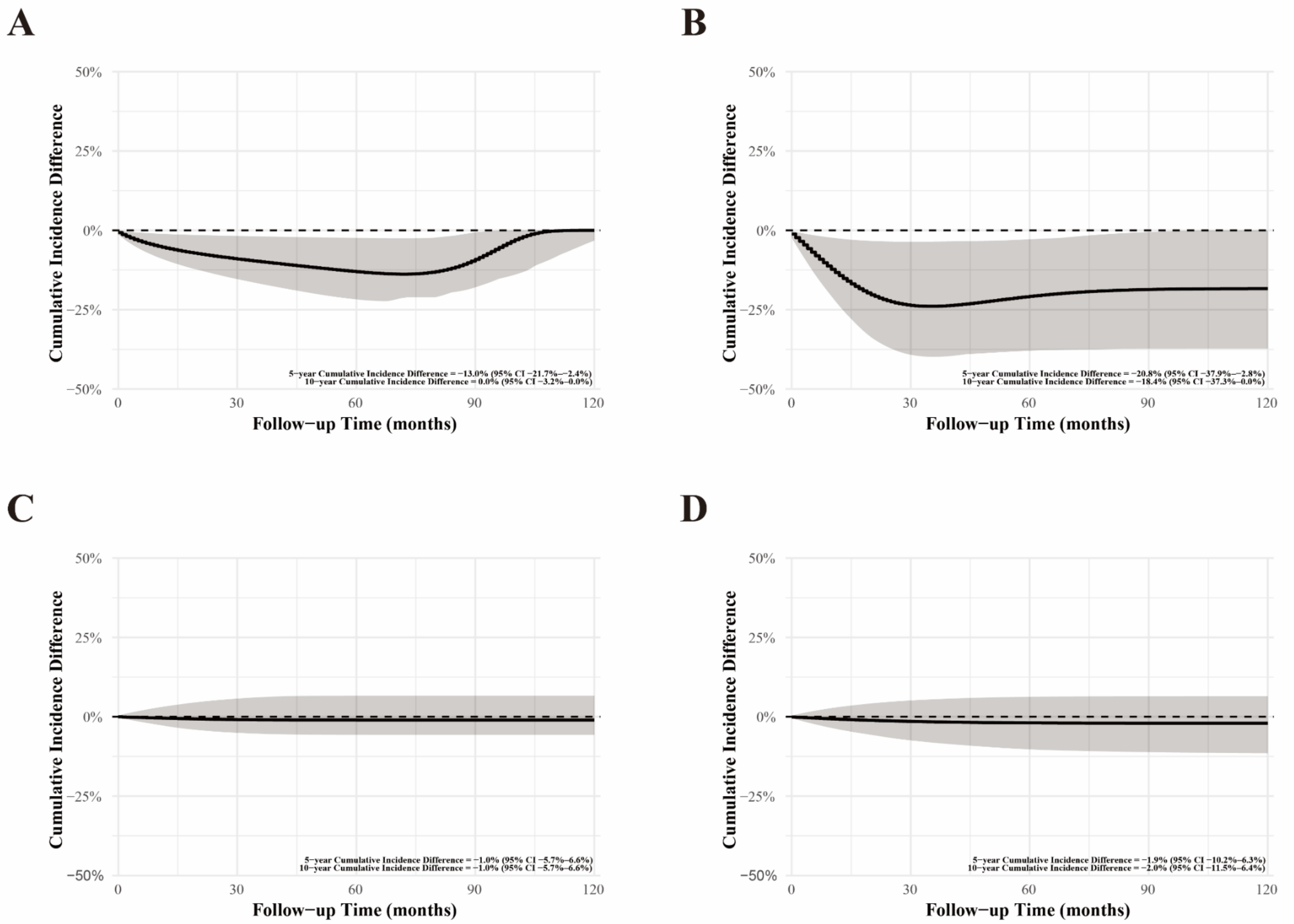

eFigure 3. Cumulative incidence difference between SGLT2 inhibitor users and non-diabetics (PP)

© 2026 Shi R et al. *JAMA Oncol.*

The difference in cumulative incidence for SGLT2 inhibitor users relative to non-diabetic subjects in the PP analysis. (**A**) time to ADT failure; (**B**) time to NHA failure; (**C**) disease-specific survival; (**D**) overall survival.

Abbreviations: SGLT2: sodium-glucose cotransporter 2; PP: per-protocol; 95% CI: 95% confidence interval.

© 2026 Shi R et al. *JAMA Oncol.*

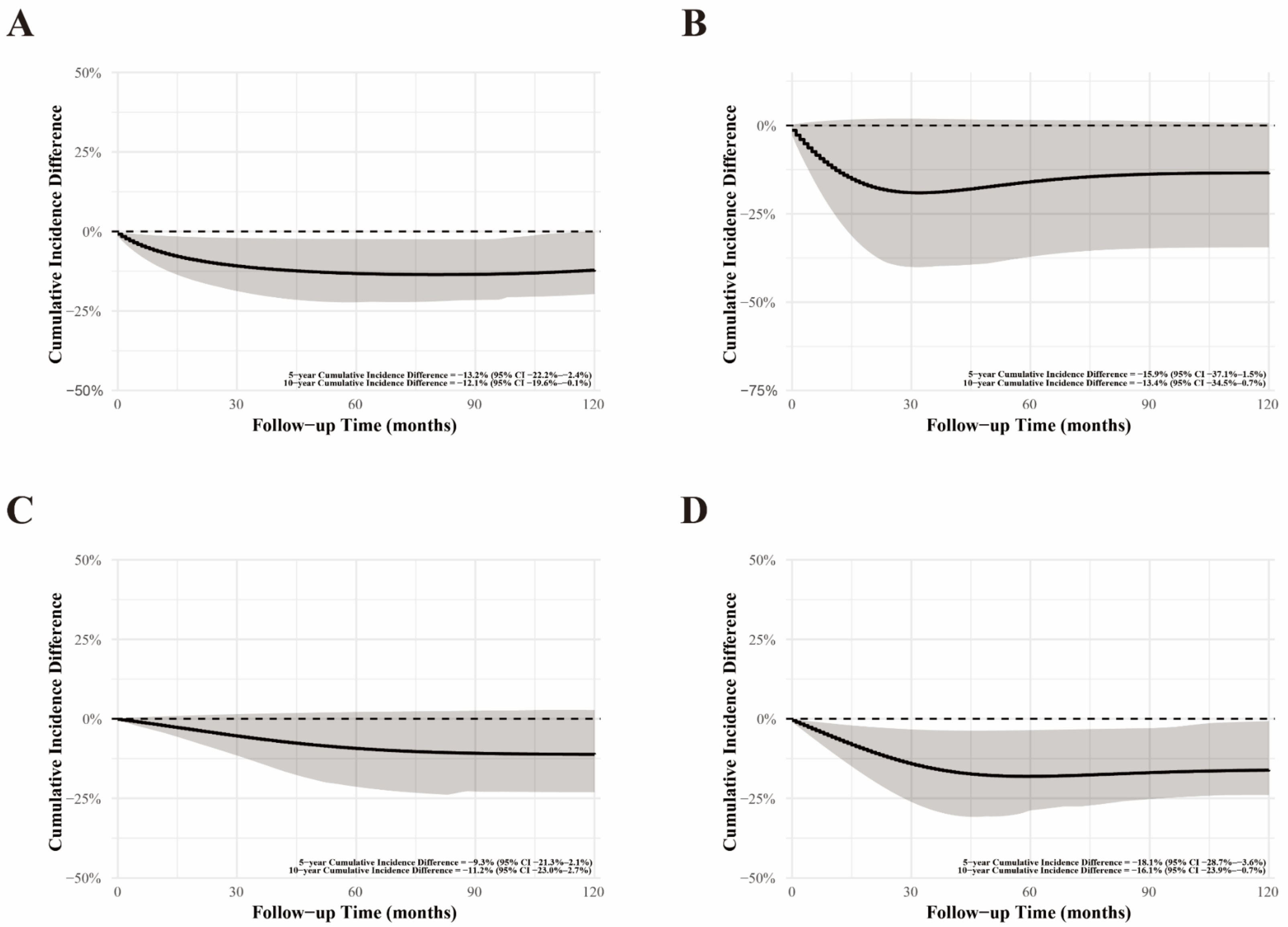

eFigure 4. Cumulative incidence difference between SGLT2 inhibitor users and subjects treated with other glucose-lowering drugs (PP)

© 2026 Shi R et al. *JAMA Oncol*.

The difference in cumulative incidence for SGLT2 inhibitor users relative to diabetic subjects treated with other glucose-lowering drugs in the PP analysis.

(**A**) time to ADT failure; (**B**) time to NHA failure; (**C**) disease-specific survival; (**D**) overall survival.

Abbreviations: SGLT2: sodium-glucose cotransporter 2; PP: per-protocol; 95% CI: 95% confidence interval.

© 2026 Shi R et al. *JAMA Oncol.*

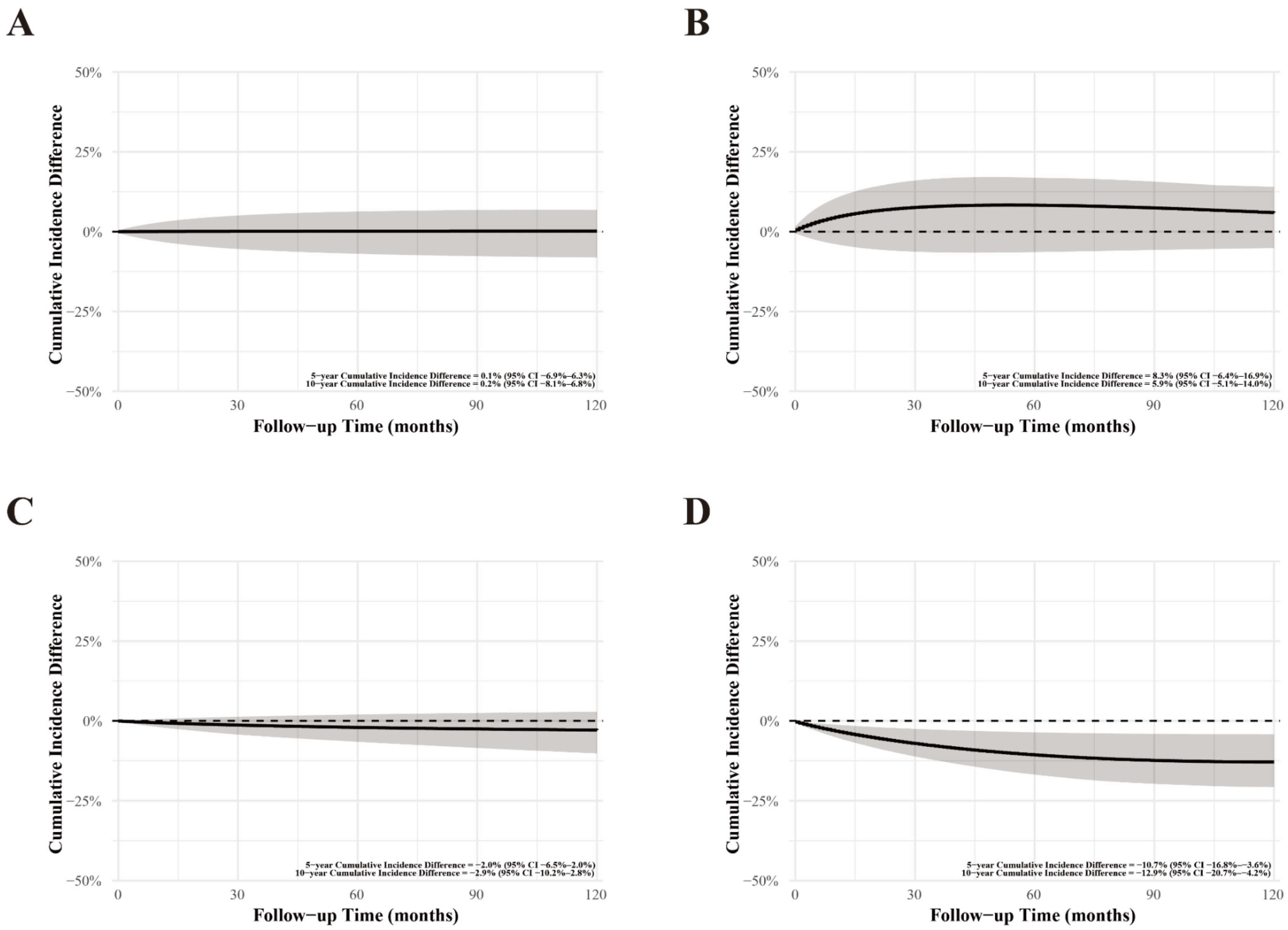

© 2026 Shi R et al. *JAMA Oncol*.

eFigure 5. Cumulative incidence difference between metformin monotherapy and non-diabetics (ITT)

The difference in cumulative incidence for metformin monotherapy users relative to non-diabetic subjects in the ITT analysis. (**A**) time to ADT failure; (**B**) time to NHA failure; (**C**) disease-specific survival; (**D**) overall survival.

Abbreviations: ITT: intention-to-treat; 95% CI: 95% confidence interval.

© 2026 Shi R et al. *JAMA Oncol.*

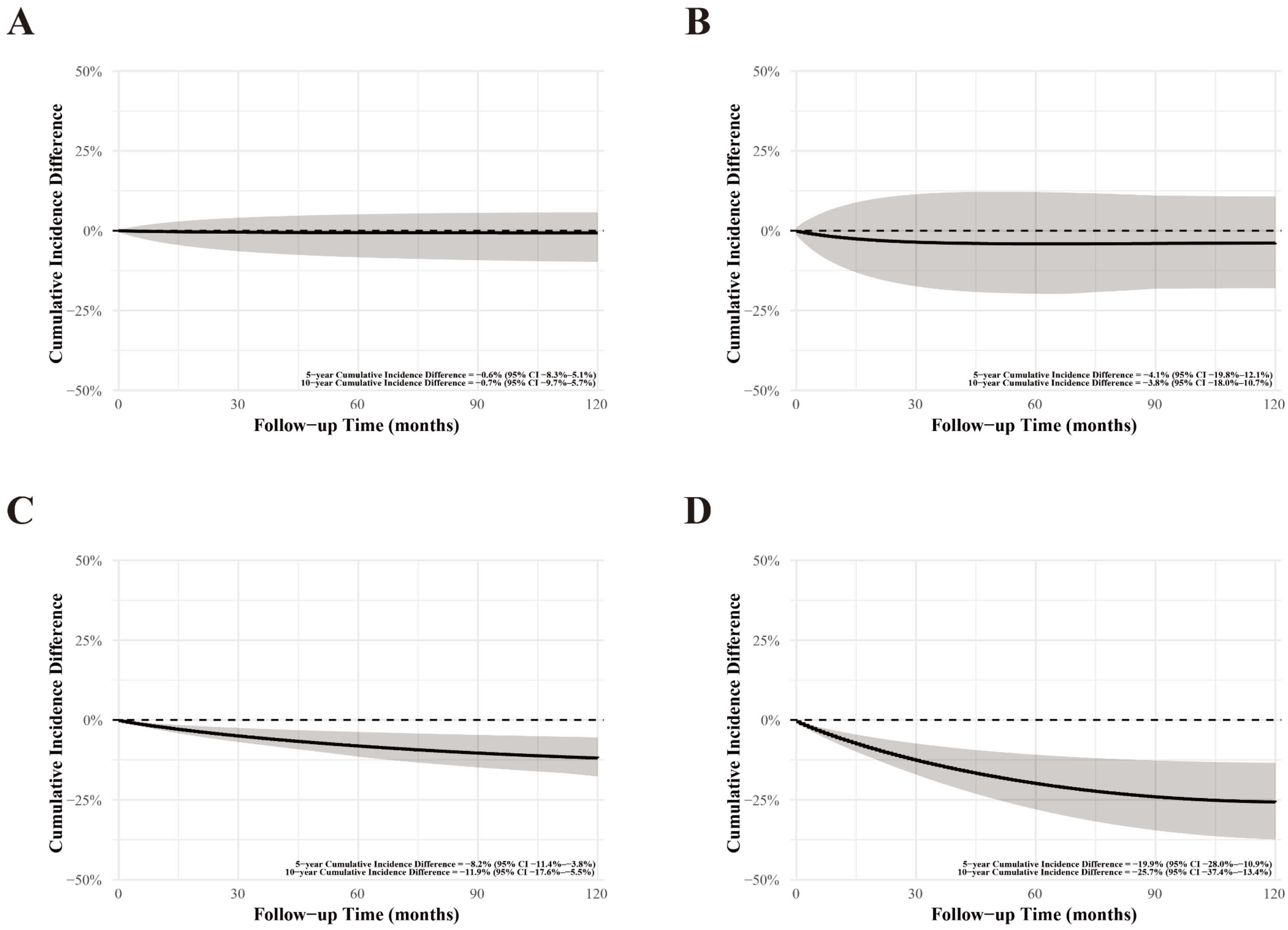

© 2026 Shi R et al. *JAMA Oncol.*

eFigure 6. Cumulative incidence difference between metformin monotherapy and non-diabetics (PP)

The difference in cumulative incidence for metformin monotherapy users relative to non-diabetic subjects in the PP analysis. (**A**) time to ADT failure; (**B**) time to NHA failure; (**C**) disease-specific survival; (**D**) overall survival.

Abbreviations: PP: per-protocol; 95% CI: 95% confidence interval.

© 2026 Shi R et al. *JAMA Oncol*.

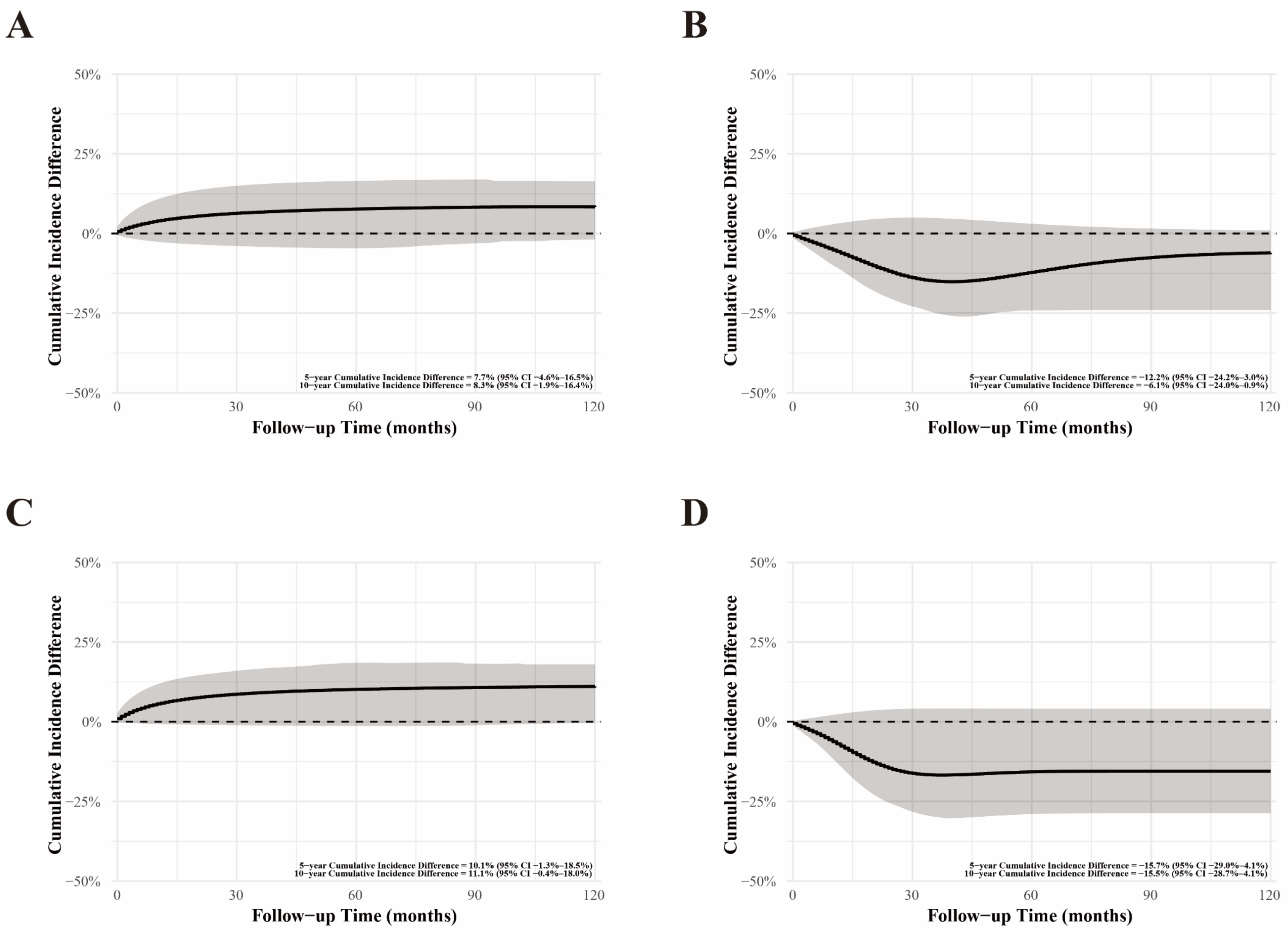

© 2026 Shi R et al. *JAMA Oncol.*

eFigure 7. Cumulative incidence difference between dapagliflozin and empagliflozin users

The difference in cumulative incidence for dapagliflozin users relative to empagliflozin users. For the intention-to-treat analysis: (**A**) time to ADT failure; (**B**) overall survival. For the per-protocol analysis: (**C**) time to ADT failure; (**D**) overall survival. 95% CI: 95% confidence interval.

© 2026 Shi R et al. *JAMA Oncol.*

eTable 4. Estimated effects of SGLT2 inhibitors and metformin on treatment failure and survival outcomes among prostate cancer patients undergoing hormone therapy (with upfront NHA included)

Intervention Group (I)	Control Group (C)	Causal Estimand	Time to ADT Failure			Time to NHA Failure		
			N (I/C)	HR (95% CI)	*P*	*N* (I/C)	HR (95% CI)	*P*
SGLT2 inhibitors	All the others	ITT	145/580	0.59 (0.37-0.93)	**.03**	30/110	0.38 (0.14-1.07)	.07
		PP		0.58 (0.35-0.95)	**.03**		0.32 (0.09-1.06)	.06
	Non-diabetic	ITT	145/580	0.59 (0.38-0.93)	**.02**	30/101	0.44 (0.13-1.41)	.16
		PP		0.51 (0.31-0.84)	**.008**		0.38 (0.08-1.66)	.19
	Other diabetic drugs	ITT	145/551	0.62 (0.42-0.94)	**.02**	30/75	0.23 (0.08-0.63)	**.004**
		PP		0.56 (0.36-0.87)	**.01**		0.18 (0.06-0.59)	**.005**
Metformin monotherapy	Non-diabetic	ITT	284/1,136	1.01 (0.76-1.34)	.97	36/144	1.37 (0.84-2.24)	.21
		PP		0.97 (0.70-1.34)	.85		0.87 (0.45-1.66)	.67
Dapagliflozin	Empagliflozin	ITT	52/112	1.76 (0.84-3.68)	.13	Results not stable.		
		PP		2.21 (0.91-5.36)	.07			

Intervention Group (I)	Control Group (C)	Causal Estimand	Disease-specific Survival			Overall Survival		
			N (I/C)	HR (95% CI)	*P*	*N* (I/C)	HR (95% CI)	*P*
SGLT2 inhibitors	All the others	ITT	146/584	0.64 (0.20-2.05)	.45	146/584	0.80 (0.45-1.41)	.43
		PP		0.70 (0.19-2.51)	.58		0.74 (0.38-1.43)	.36
	Non-diabetic	ITT	146/584	1.02 (0.42-2.50)	.95	146/584	0.97 (0.59-1.58)	.90
		PP		0.96 (0.30-3.07)	.95		0.85 (0.46-1.59)	.61
	Other diabetic drugs	ITT	146/560	0.42 (0.15-1.15)	.09	146/560	0.55 (0.34-0.89)	**.02**
		PP		0.39 (0.12-1.27)	.12		0.43 (0.25-0.77)	**.004**
Metformin monotherapy	Non-diabetic	ITT	287/1,148	0.74 (0.46-1.19)	.21	287/1,148	0.58 (0.41-0.81)	**.001**
		PP		0.29 (0.13-0.61)	**.001**		0.34 (0.21-0.54)	**<.001**
Dapagliflozin	Empagliflozin	ITT	Results not stable.			52/112	0.23 (0.06-0.96)	**.04**
		PP					0.13 (0.01-1.22)	.07

Abbreviations: SGLT2: sodium-glucose cotransporter 2; NHA: next-generation hormonal agent; ADT: androgen deprivation therapy; HR: hazard ratio; 95% CI: 95% confidence interval; ITT: intention-to-treat; PP: per-protocol. A two-sided *P*-value <0.05 was considered statistically significant.

© 2026 Shi R et al. *JAMA Oncol.*

eTable 5. Sensitivity analysis of SGLT2 inhibitors on treatment failure and survival outcomes among prostate cancer patients undergoing hormone therapy with an ADT grace period of 12 months

Intervention Group (I)	Control Group (C)	Causal Estimand	Time to ADT Failure			Time to NHA Failure		
			***N* (I/C)**	**HR (95% CI)**	***P***	***N* (I/C)**	**HR (95% CI)**	***P***
SGLT2 inhibitors	All the others	ITT	160/640	0.55 (0.36-0.84)	**.006**	45/175	0.51 (0.24-1.08)	.08
		PP		0.53 (0.33-0.83)	**.005**		0.50 (0.22-1.15)	.10
	Non-diabetic	ITT	160/640	0.66 (0.44-1.01)	.05	45/161	0.40 (0.18-0.88)	**.02**
		PP		0.58 (0.37-0.93)	**.02**		0.46 (0.18-1.15)	.10
	Other diabetic drugs	ITT	160/593	0.62 (0.42-0.91)	**.02**	44/130	0.53 (0.24-1.17)	.12
		PP		0.59 (0.39-0.90)	**.01**		0.58 (0.24-1.39)	.22

Intervention Group (I)	Control Group (C)	Causal Estimand	Disease-specific Survival			Overall Survival		
			***N* (I/C)**	**HR (95% CI)**	***P***	***N* (I/C)**	**HR (95% CI)**	***P***
SGLT2 inhibitors	All the others	ITT	161/644	0.42 (0.15-1.19)	.10	161/644	0.72 (0.44-1.19)	.20
		PP		0.45 (0.14-1.43)	.18		0.66 (0.37-1.19)	.16
	Non-diabetic	ITT	161/644	1.03 (0.46-2.35)	.93	161/644	0.89 (0.56-1.44)	.65
		PP		1.06 (0.37-3.04)	.92		0.90 (0.50-1.62)	.72
	Other diabetic drugs	ITT	161/602	0.53 (0.22-1.25)	.15	161/602	0.63 (0.40-0.99)	**.04**
		PP		0.43 (0.15-1.23)	.11		0.49 (0.28-0.83)	**.009**

Abbreviations: SGLT2: sodium-glucose cotransporter 2; ADT: androgen deprivation therapy; NHA: next-generation hormonal agent; HR: hazard ratio; 95% CI: 95% confidence interval; ITT: intention-to-treat; PP: per-protocol. A two-sided *P*-value <0.05 was considered statistically significant.

© 2026 Shi R et al. *JAMA Oncol.*

eTable 6. Sensitivity analysis of SGLT2 inhibitors on treatment failure and survival outcomes among prostate cancer patients undergoing hormone therapy with varying SGLT2 inhibitor grace periods

Intervention Group (I)	Control Group (C)	Causal Estimand	Time to ADT Failure			Time to NHA Failure		
			N (I/C)	HR (95% CI)	*P*	*N* (I/C)	HR (95% CI)	*P*
SGLT2 inhibitors *(Grace period = 30 days)*	All the others	ITT	158/632	0.65 (0.43-0.98)	**.04**	44/171	0.43 (0.19-0.96)	**.04**
		PP		0.62 (0.40-0.97)	**.04**		0.45 (0.18-1.12)	.08
SGLT2 inhibitors *(Grace period = 180 days)*	All the others	ITT	161/644	0.58 (0.38-0.87)	**.009**	45/175	0.45 (0.20-0.97)	**.04**
		PP		0.56 (0.36-0.87)	**.01**		0.45 (0.19-1.10)	.08

Intervention Group (I)	Control Group (C)	Causal Estimand	Disease-specific Survival			Overall Survival		
			N (I/C)	HR (95% CI)	*P*	*N* (I/C)	HR (95% CI)	*P*
SGLT2 inhibitors *(Grace period = 30 days)*	All the others	ITT	159/636	0.52 (0.18-1.52)	.23	159/636	0.75 (0.45-1.26)	.28
		PP		0.57 (0.17-1.92)	.36		0.69 (0.38-1.25)	.22
SGLT2 inhibitors *(Grace period = 180 days)*	All the others	ITT	162/648	0.50 (0.18-1.43)	.20	162/648	0.85 (0.52-1.40)	.52
		PP		0.55 (0.17-1.82)	.33		0.73 (0.41-1.31)	.29

Abbreviations: SGLT2: sodium-glucose cotransporter 2; ADT: androgen deprivation therapy; NHA: next-generation hormonal agent; HR: hazard ratio; 95% CI: 95% confidence interval; ITT: intention-to-treat; PP: per-protocol. A two-sided *P*-value <0.05 was considered statistically significant.

© 2026 Shi R et al. *JAMA Oncol.*

eTable 7. Sensitivity analyses of SGLT2 inhibitors on treatment failure and survival outcomes among prostate cancer patients undergoing hormone therapy using conventional Cox regression model

Intervention Group (I)	Control Group (C)	Time to ADT Failure			Time to NHA Failure		
		N (I/C)	HR (95% CI)	*P*	*N* (I/C)	HR (95% CI)	*P*
SGLT2 inhibitors	All the others	303/1,212	0.36 (0.27-0.48)	**<.001**	92/363	0.50 (0.32-0.79)	**.003**
	Non-diabetic	303/1,212	0.33 (0.23-0.47)	**<.001**	92/343	0.39 (0.22-0.69)	**.001**
	Other diabetic drugs	303/1,186	0.30 (0.23-0.39)	**<.001**	91/277	0.37 (0.24-0.58)	**<.001**

Intervention Group (I)	Control Group (C)	Disease-specific Survival			Overall Survival		
		N (I/C)	HR (95% CI)	*P*	*N* (I/C)	HR (95% CI)	*P*
SGLT2 inhibitors	All the others	411/1,642	0.34 (0.22-0.52)	**<.001**	411/1,642	0.40 (0.31-0.52	**<.001**
	Non-diabetic	411/1,637	0.47 (0.28-0.78)	**.003**	411/1,637	0.46 (0.34-0.63)	**<.001**
	Other diabetic drugs	411/1,541	0.36 (0.24-0.53)	**<.001**	411/1,541	0.39 (0.30-0.49)	**<.001**

Abbreviations: SGLT2: sodium-glucose cotransporter 2; ADT: androgen deprivation therapy; NHA: next-generation hormonal agent; HR: hazard ratio; 95% CI: 95% confidence interval. A two-sided *P*-value <0.05 was considered statistically significant.

© 2026 Shi R et al. *JAMA Oncol.*
