# Supplementary material for: Sodium-Glucose Cotransporter 2 Inhibitors for Patients With Prostate Cancer Undergoing Hormone Therapy
Source: JAMA Oncol. 2026 Jan 8;12(3):257–65. doi: 10.1001/jamaoncol.2025.5869 (PMC12784257; doi:10.1001/jamaoncol.2025.5869)
Supplement: Supplement 2. — Data Sharing Statement [file jamaoncol-e255869-s002.pdf]

Data Sharing Statement

Shi. Sodium-Glucose Cotransporter 2 Inhibitors for Patients With Prostate Cancer Undergoing Hormone Therapy. *JAMA Oncol*. Published January 08, 2026.
doi:10.1001/jamaoncol.2025.5869

Data
Data available: No
